# Magnetic resonance neuroimaging in patients from a psychiatric facility

**DOI:** 10.4102/sajpsychiatry.v24i0.1283

**Published:** 2018-09-26

**Authors:** Vidette Juby, Saeeda Paruk

**Affiliations:** 1 Department of Psychiatry, University of KwaZulu-Natal, South Africa

## Abstract

**Background:**

Many neurological conditions manifest with psychiatric symptoms and may be misdiagnosed. Structural neuroimaging, that is, computerised tomography (CT) and magnetic resonance imaging (MRI), can aid in the diagnosis or exclusion of these conditions. MRI is preferable in this regard but is more expensive and less readily available than CT. However, the indications for the requisitions of MRI in the clinical psychiatric setting remain poorly defined. All published literature on the clinical utility of neuroimaging in Africa is on CT scans.

**Aim:**

To describe the clinical characteristics and MRI findings in a cohort of patients presenting with psychiatric symptoms.

**Methods:**

Retrospective chart review of all patients who underwent MRI between 01 October 2010 and 31 June 2016. MRI findings were correlated with socio-demographic and clinical information including psychiatric diagnosis, indication for MRI imaging and impact on patient management.

**Results:**

Of 53 MRIs performed, 33 (62%) were abnormal. Patients with HIV, neurocognitive disorders, chronic mental illness and involuntary admission were more likely to have abnormal scans (83%, 80%, 71% and 79%, respectively); 54% of abnormal MRIs (24% of all MRIs performed) necessitated referral to other disciplines. No statistically significant associations were found with socio-demographic or clinical factors.

**Conclusion:**

Abnormalities on MRI scans in mentally ill patients were common and a quarter required referral to other disciplines. Further studies are required to clarify the clinical utility of MRI in patients with psychiatric illness which could assist in the development of a guide to the rational use of this modality in a resource-constrained environment.

